# Uses of a real-time automatic nosocomial infection surveillance system to support prevention and control of hospital-acquired infections in the ICU

**DOI:** 10.3389/fpubh.2024.1399067

**Published:** 2024-09-13

**Authors:** Xinying Li, Peihong Cai, Huiting Zhong, Caili Yan, Ruiling Wen

**Affiliations:** Department of Infection Management, Huizhou First Hospital, Huizhou, Guangdong, China

**Keywords:** hospital-acquired infections, intensive care unit, infection surveillance, infection prevention and control, device-associated infections

## Abstract

**Introduction:**

The intensive care unit (ICU) caters to patients with severe illnesses or injuries who require constant medical attention. These patients are susceptible to infections due to their weak immune systems and prolonged hospital stays. This makes the ICU the specialty with the highest hospital-acquired infection (HAI) cases. The core dimension of infection prevention and control for ICUs is infection surveillance, which analyses the risk factors of HAI and implements comprehensive interventions for HAI prevention and control. Hence, this study aimed to investigate the potential risk factors for developing HAI in the ICU using real-time automatic nosocomial infection surveillance systems (RT-NISS) to surveil, and analyze the effectiveness of RT-NISS coupled with comprehensive interventions on HAI prevention and control in the ICU.

**Methods:**

A retrospective analysis was conducted using data from an RT-NISS for all inpatients in the ICU from January 2021 to December 2022. Univariate and multivariate logistic regression analyses were performed to analyse potential risk factors for HAI in the ICU. Surveillance of the prevalence proportion of HAI, the prevalence proportion of site-specific HAI, the proportion of ICU patients receiving antibiotics, the proportion of ICU patients receiving key antimicrobial combination, the proportion of HAI patients with pathogen detection, the proportion of patients with pathogen detection before antimicrobial treatment and the proportion of patients before receiving key antimicrobial combination, the utilization rate of devices and the rate of device-associated HAIs were monitored monthly by the RT-NISS. Comprehensive interventions were implemented in 2022, and we compared the results of HAIs between 2021 and 2022 to evaluate the effect of the RT-NISS application combined with comprehensive interventions on HAI prevention and control.

**Results:**

The relative risk factors, observed as being a significantly higher risk of developing HAI, were hospitalization over 2 weeks, chronic lung diseases, chronic heart diseases, chronic renal diseases, current malignancy, hypohepatia, stroke, cerebrovascular accident, severe trauma, tracheal intubation and tracheostomy and urinary catheter. By implementing comprehensive interventions depending on infection surveillance by the RT-NISS in 2022, the prevalence proportion of HAI was reduced from 12.67% in 2021 to 9.05% in 2022 (χ^2^ = 15.465, *p* < 0.001). The prevalence proportion of hospital-acquired multidrug-resistant organisms was reduced from 5.78% in 2021 to 3.21% in 2022 (χ^2^ = 19.085, *p* < 0.001). The prevalence proportion of HAI in four sites, including respiratory tract infection, gastrointestinal tract infection, surgical site infection, and bloodstream infection, was also significantly reduced from 2021 to 2022 (both *p* < 0.05). The incidence of ventilator-associated pneumonia in 2022 was lower than that in 2021 (15.02% vs. 9.19%, χ^2^ = 17.627, *p* < 0.001).

**Conclusion:**

The adoption of an RT-NISS can adequately and accurately collect HAI case information to analyse the relative high-risk factors for developing HAIs in the ICU. Furthermore, implementing comprehensive interventions derived from real-time automation surveillance of the RT-NISS will reduce the risk and prevalence proportions of HAIs in the ICU.

## 1 Introduction

Intensive care units (ICUs) have the highest prevalence proportion of hospital-acquired infections (HAIs) due to patients’ weakened immune systems and the prevalence of more invasive medical procedures (1–4). This makes the ICU the specialty unit with the highest concentration of patients with high-risk HAIs, making surveillance of HAIs a top priority (1, 4). Surveillance and feedback on HAIs to promptly identify patients at risk and enable the implementation of targeted infection control measures such as hand hygiene, isolation precautions, and environmental sanitation are the fundamental elements essential to prevent the spread of pathogens within the ICU and for infection prevention and control programs to be effective (5–9). However, traditional surveillance by infection control physicians, which involves manually reviewing the medical records of all patients at risk for HAI, is highly resource-intensive and cannot be delivered in a timely and efficient manner, leading to delayed implementation of appropriate interventions (8, 10). Therefore, real-time automatic nosocomial infection surveillance systems (RT-NISSs) have been developed and applied. RT-NISSs are reliable surveillance intelligent information technology tools that enable infection control physicians to systematically and continuously monitor and evaluate HAI-related information, including patient data, laboratory results, and other relevant information, to identify trends and patterns indicative of potential infections in a timely, adequate, and accurate manner (10–14). Once infection control physicians or clinicians identify HAI cases using the RT-NISS, they can take strategies and measures to control such cases immediately (15).

In this study, we describe and analyse the effect of the adoption of an RT-NISS for real-time monitoring of HAI trends, coupled with timely corresponding interventions on HAI prevention and control in the ICU.

## 2 Materials and methods

### 2.1 Study setting and data collection

We conducted a retrospective study of all patients admitted to the ICU of Huizhou First Hospital (HZFH) for >48 h from January 2021 to December 2022. An RT-NISS (Xinglin Technology, Hangzhou, China) was used to monitor all inpatients during their hospital stay in the ICU.

The RT-NISS used in our study was systematically applied to monitor, diagnose, and control HAIs. It was activated, captured patients’ infection information from other hospital information systems including the hospital information system (HIS), LIS, radiology information system (RIS) and anaesthesia operation system (AOS) and provided new HAIs alert at 2:00 am every day. It can real-time automately, adequately and accurately collect inpatient information and selects patients with the highest HAI probability by setting a filter to acquire the necessary infection information (e.g., temperature, serological and molecular testing results, diagnostic test results, microbiology laboratory results, prescription of antibiotic, radiology examination results and so on). Infection control physicians and clinicians would obtain infection information and make judgment and final diagnostic by using the RT-NISS (12).

Patients admitted to the ICU of HZFH for >48 h from January 2021 to December 2022 were categorized into two groups (the HAI group and Non-HAI group) to analyze the potential risk factors of HAI in the ICU. Data on 18 potential risk factors included age, male, hospitalization over 2 weeks, antimicrobial prophylaxis, comorbidities, chronic lung diseases, chronic heart diseases, chronic renal diseases, current malignancy, hypohepatia, hypertension, diabetes mellitus, stroke, cerebral vascular accident, severe trauma, surgical intervention, tracheal intubation and tracheostomy, urinary catheter and CVC were collected by the RT-NISS and analysed by univariate analysis. And potential risk factors considered statistically significant by univariate analysis were analysed by multivariate logistic regression analysis. Data on the incidence of device-associated infections (central line-associated bloodstream infection [CLABSI], ventilator-associated pneumonia [VAP] and catheter-associated UTI [CAUTI]) of the catheter at utilization over 1 week, 2 weeks and 1 month, respectively, was collected by the RT-NISS and analysed the effect of the length of catheter utilization time on the incidence of device-associated infections.

Patients admitted to the ICU of HZFH for >48 h from January 2021 to December 2022 were categorized into two groups: the 2021 group included patients from January 2021 to December 2021 and the 2022 group included patients from January 2022 to December 2022. Data collected by the RT-NISS included the prevalence proportion of HAI, the prevalence proportion of site-specific HAI (respiratory infection, UTI, GTI, SSI, BSI, abdominal infection, encephalitis skin and soft tissue infection), the proportion of ICU patients receiving antibiotics, the proportion of ICU patients receiving key antimicrobial combination, the proportion of HAI patients with pathogen detection, the proportion of patients with pathogen detection before antimicrobial treatment and the proportion of patients with pathogen detection before receiving key antimicrobial combination of two group were analysed and compared.

### 2.2 Comprehensive interventions

In this study, comprehensive interventions implemented in the ICU included the following: (1) Risk factors for specific infections (e.g., urinary tract infection [UTI], sepsis, pneumonia, and device-associated HAIs), high infection sites or infection clusters (the same pathogens ≥3 within 7 days in a single ward) were analysed using quality management tools, and appropriate corresponding improvement measures, including increasing environmental hygiene monitoring, supervision of hand hygiene, strengthening environmental hygiene and sufficient training for healthcare workers, were formulated. (2) Multidrug-resistant organism (MDRO) case transmission and prevention measures, including contact isolation, isolation with personal protective equipment, and hand and environmental hygiene, were stringently implemented. (3) Active screening tests include respiratory specimen microbiological examination for patients with severe pneumonia, routine urine tests or the microbiological examination of other infection sites implemented before or on the first day of patient admission. (4) Increasing pathogen detection can promote the rational use of antibiotics and detect potential infections in a timely manner. With the consideration of these, we pay attention to surveilling of the proportion of ICU patients receiving antibiotics, the proportion of ICU patients receiving key antimicrobial combination, the proportion of HAI patients with pathogen detection, the proportion of patients with pathogen detection before antimicrobial treatment and the proportion of patients with pathogen detection proportion of patients before receiving key antimicrobial combination (imipenem, meropenem, panipenem, biapenem, ertapenem, vancomycin, teicoplanin, tigecycline, linezolid, polymyxin, cefoperazone sulbactam, voriconazole, itraconazole, capafungin) were monitored by the RT-NISS. Pathogen detection in this study involved microbiological examinations, pathogen microscope tests, pathogen polymerase chain reaction detection, serological calcitonin tests, serological interleukin-6 (IL-6) tests or fungus (1–3)-β-D-glucan tests. (5) Target surveillance of device utilization rates (ventilator, urinary catheter and central venous catheter [CVC]) and the incidence of device-associated infections (VAP, CAUTI, and CLABSI) were monitored by the RT-NISS. (6) Sufficient variable interval training included adequate hand hygiene, environment hygiene, reasonable use of antibiotics, pathogen detection before antimicrobial treatment and prevention and control knowledge of MDRO and device-associated infections were provided to healthcare workers (physicians, nurses, and cleaners).

### 2.3 Definitions

We defined HAIs according to the Nosocomial Infection Diagnostic Criteria in 2001 published by the Ministry of Public Health of the People’s Republic of China (16). HAIs are infections acquired >48 h after hospital admission and not present and incubated at the time of admission. According to the specific infection site, HAIs are classified as respiratory infection, UTI, gastrointestinal tract infection (GTI), surgical site infection (SSI), bloodstream infection (BSI), abdominal infection, encephalitis skin and soft tissue infection and other infections. Device-associated HAIs (VAP, CAUTI, and CLABSI) were those infections after >48 h of device application or <48 h after extubation. MDROs, including extended-spectrum beta-lactamase-producing gram-negative bacilli, carbapenem-resistant Enterobacterales, methicillin-resistant *Staphylococcus aureus*, vancomycin-resistant enterococci, carbapenem-resistant *Acinetobacter baumannii*, and carbapenem-resistant *Pseudomonas aeruginosa*, were studied.

### 2.4 Data analysis

All statistical analyses were performed by using IBM SPSS Statistics version 22.0 software (IBM Corp., Armonk, N.Y., USA). Descriptive statistics were used to calculate the prevalence proportion of HAI, the prevalence proportion of site-specific HA (respiratory infection, UTI, GTI, SSI, BSI, abdominal infection, encephalitis skin and soft tissue infection), the proportion of ICU patients receiving antibiotics, the proportion of ICU patients receiving key antimicrobial combination, the proportion of HAI patients with pathogen detection, the proportion of patients with pathogen detection before antimicrobial treatment and the proportion of patients with pathogen detection proportion of patients with pathogen detection before receiving key antimicrobial combination:

the prevalence proportion of HAI $=\frac{\mathrm{the}\;\mathrm{number}\;\mathrm{of}\;HAI\;\mathrm{in}\;\mathrm{the}\;ICU}{\mathrm{the}\;\mathrm{number}\;\mathrm{of}\;\mathrm{hospital}\;\mathrm{admissions}>48h\;\mathrm{in}\;\mathrm{the}\;ICU}*100\%\text{.}$

the prevalence proportion of site-specific HAI $=\frac{\mathrm{the}\;\mathrm{number}\;\mathrm{of}\;\mathrm{the}\;\mathrm{specific}\;\mathrm{infective}\;\mathrm{site}\;\mathrm{in}\;\mathrm{the}\;ICU}{\mathrm{the}\;\mathrm{number}\;\mathrm{of}\;\mathrm{hospital}\;\mathrm{admissions}>48h\;\mathrm{in}\;\mathrm{the}\;ICU}*100\%$

the incidence of device-associated infection $=\frac{\begin{array}{l} \mathrm{the}\;\mathrm{number}\;\mathrm{of}\;\mathrm{device}-\mathrm{associated}\;\mathrm{infection}\;\mathrm{with}\;\\ \mathrm{catheter}\;\mathrm{at}\;\mathrm{utilization}\;\mathrm{over}\;1\mathrm{week}/2\mathrm{weeks}/1\mathrm{month} \end{array}}{\mathrm{the}\;\mathrm{total}\;\mathrm{number}\;\mathrm{of}\;\mathrm{device}-\mathrm{associated}\;\mathrm{infection}}*1000‰$

the proportion of ICU patients receiving antibiotics $=\frac{\mathrm{the}\;\mathrm{number}\;\mathrm{of}\;\mathrm{patients}\;\mathrm{receive}\;\mathrm{antibiotics}}{\mathrm{the}\;\mathrm{number}\;\mathrm{of}\;\mathrm{hospital}\;\mathrm{admissions}>48h\;\mathrm{in}\;\mathrm{the}\;ICU}*100\%$

the proportion of ICU patients receiving key antimicrobial combination $=\frac{\mathrm{the}\;\mathrm{number}\;\mathrm{of}\;\mathrm{patients}\;\mathrm{receive}\;\mathrm{key}\;\mathrm{antimicrobial}\;\mathrm{combination}}{\mathrm{the}\;\mathrm{number}\;\mathrm{of}\;\mathrm{patients}\;\mathrm{receive}\;\mathrm{antibiotics}}*100\%$

the proportion of HAI patients with pathogen detection $=\frac{\mathrm{the}\;\mathrm{number}\;\mathrm{of}\;HAI\;\mathrm{patients}\;\mathrm{performed}\;\mathrm{pathogen}\;\mathrm{detection}}{\mathrm{the}\;\mathrm{number}\;\mathrm{of}\;HAI}*100\%$

the proportion of patients with pathogen detection before antimicrobial treatment $=\frac{\begin{array}{l} \mathrm{the}\;\mathrm{number}\;\mathrm{of}\;\mathrm{patients}\;\mathrm{performed}\;\mathrm{pathogen}\;\\ \mathrm{detection}\;\mathrm{before}\;\mathrm{antimicrobial}\;\mathrm{treatment} \end{array}}{\mathrm{the}\;\mathrm{number}\;\mathrm{of}\;\mathrm{patients}\;\mathrm{receive}\;\mathrm{antibiotics}}*100\%$

the proportion of patients with pathogen detection before receiving key antimicrobial combination $=\frac{\begin{array}{l} \mathrm{the}\;\mathrm{number}\;\mathrm{of}\;\mathrm{patients}\;\mathrm{performed}\;\mathrm{pathogen}\;\mathrm{detection}\;\\ \mathrm{before}\;\mathrm{treatment}\;\mathrm{with}\;\mathrm{key}\;\mathrm{antimicrobial}\;\mathrm{combination} \end{array}}{\mathrm{the}\;\mathrm{number}\;\mathrm{of}\;\mathrm{patients}\;\mathrm{with}\;\mathrm{key}\;\mathrm{antimicrobial}\;\mathrm{combination}}*100\%$

Incidences of device-associated infections, including VAP, CAUTI, and CVC, were calculated per 1,000 device days. Either the χ^2^-test or Fisher’s exact test was used for the comparison of categorical variables. Multivariate regression analysis using the logistics regression model was used to calculate the corresponding regression coefficients: the Chi-square test of inferential statistics was used in univariate analysis to compare potential risk factors data between the HAI group and the Non-HAI group. The potential risk factors considered statistically significant by univariate analysis were independent variables and whether nosocomial infection occurred 48 h after admission was used as the dependent variable. Two-tailed *p* values <0.05 were considered statistically significant and had a 95% confidence interval (CI).

### 2.5 Ethics approval and consent to participate

This study was reviewed and approved by the Ethics Committee of Huizhou First Hospital. The ethics committee approved the waiver of informed consent, given the retrospective nature of the review. All patient records were confirmed eligible for collection in accordance with the relevant guidelines and regulations.

## 3 Results

### 3.1 The potential risk factors for developing HAI in the ICU

#### 3.1.1 Univariate analysis of potential risk factors for HAI in the ICU

We used the RT-NISS to collect the data of 3,475 patients (1706 patients in 2021 and 1769 in 2022) admitted to the ICU of HZFH after >48 h from January 2021 to December 2022, including 607 (17.47%) HAI cases. The numbers and rates of potential risk factors for HAI in the univariate analysis are shown in Table 1. Compared with non-HAI, the risk of developing HAI increased with age ≥ 60 years, hospitalization over 2 weeks, antimicrobial prophylaxis, chronic lung diseases, chronic heart diseases, chronic renal diseases, current malignancy, hypohepatia, hypertension, stroke, cerebral vascular accident, severe trauma, surgical intervention, tracheal intubation and tracheostomy, urinary catheter, and CVC. Our study also showed that more patients with HAI died than those without HAI (67 (11.04%) vs. 174 (6.07%), χ^2^ = 19.180, *p* < 0.001).

**Table 1:** Univariate analysis of potential risk factors for HAI in the ICU.

Comparison	HAI (*n* = 607)	Non-HAI (*n* = 2,868)	χ^2^	*P*
Age ≥ 60 years (*n*/%)	291 (47.94)	1,186 (41.35)	8.897	0.003
Male (*n*/%)	417 (68.70)	1871 (65.24)	2.669	0.102
Hospitalization over 2 weeks (*n*/%)	368 (60.63)	439 (15.31)	577.069	<0.001
Antimicrobial prophylaxis (*n*/%)	392 (64.58)	942 (32.85)	213.314	<0.001
Comorbidities (*n*/%)				
Chronic lung diseases	187 (30.81)	479 (16.70)	64.341	<0.001
Chronic heart diseases	83 (13.67)	301 (10.50)	5.150	0.023
Chronic renal diseases	55 (9.06)	105 (3.66)	33.257	<0.001
Current malignancy	37 (6.10)	112 (3.91)	5.857	0.016
Hypohepatia	53 (8.73)	164 (5.72)	7.769	0.005
Hypertension	225 (37.07)	901 (31.42)	7.306	0.007
Diabetes mellitus	102 (16.80)	409 (14.26)	2.583	0.108
Stroke	108 (17.79)	192 (6.69)	78.223	<0.001
Cerebral vascular accident	231 (38.06)	597 (20.82)	82.039	<0.001
Severe trauma	192 (31.63)	673 (23.47)	17.865	<0.001
Surgical intervention	409 (67.38)	1,023 (35.67)	207.938	<0.001
Catheter at admission (*n*/%)				
Tracheal intubation and tracheostomy	528 (86.99)	1,217 (42.43)	397.745	<0.001
Urinary catheter	601 (99.01)	1929 (67.26)	255.103	<0.001
CVC	508 (83.69)	1,244 (43.38)	325.716	<0.001

HAI, hospital-acquired infection; ICU, intensive care unit; CVC, central venous catheter.

#### 3.1.2 Multivariate logistic regression analysis of potential risk factors for HAI in the ICU

**Figure 1:**
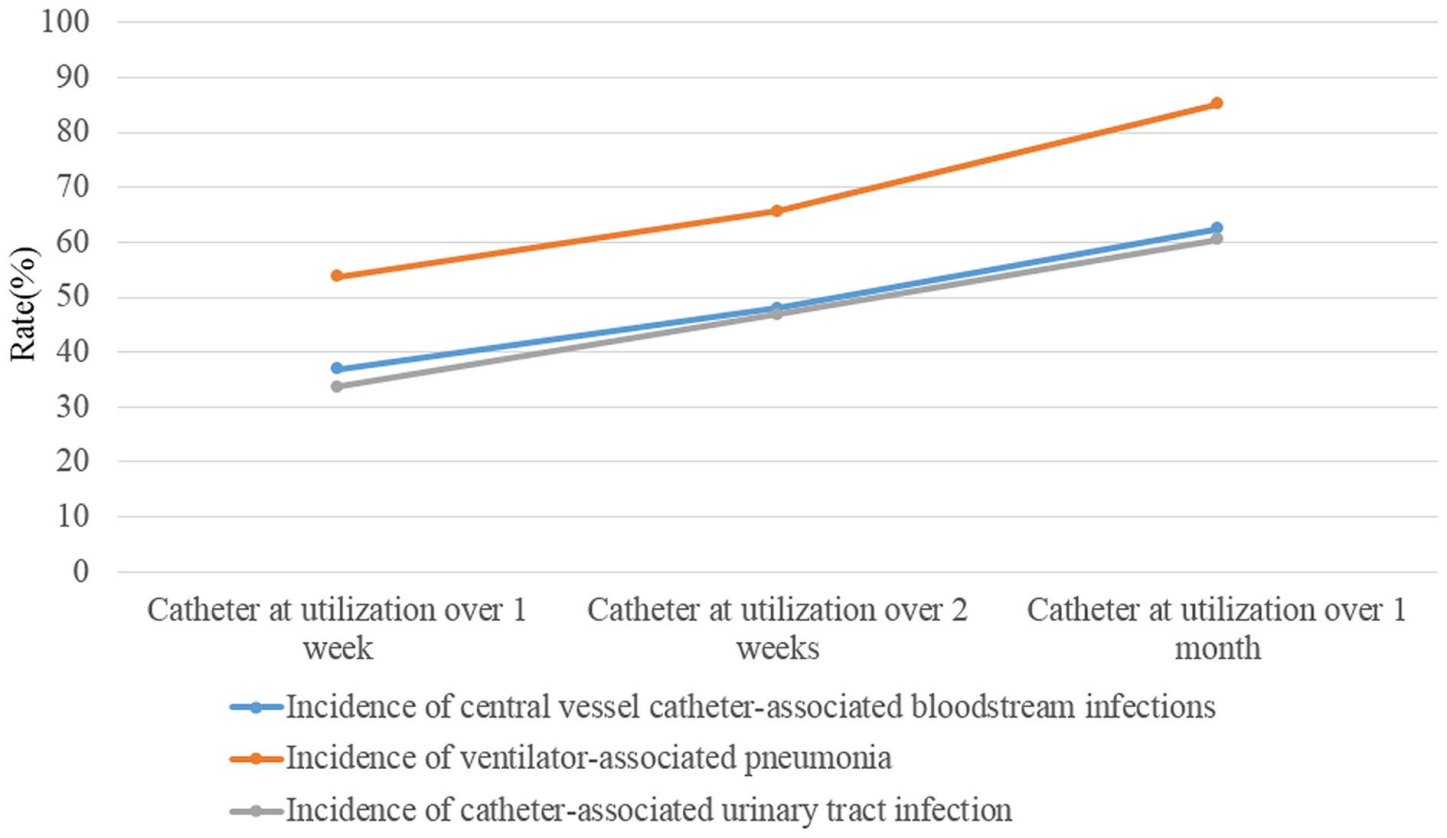
The incidences of device-associated HAIs (ventilator-associated pneumonia, catheter-associated urinary tract infection, and central line-associated bloodstream infection) after catheter (ventilator, urinary catheter and central venous catheter) utilization over 1 week, 2 weeks and 1 month.

Table 2 shows the results of multivariate logistic regression analysis according to the results of univariate analysis. Hospitalization over 2 weeks, patients with: chronic lung diseases, chronic heart diseases, chronic renal diseases, current malignancy, hypohepatia, stroke, cerebral vascular accident, severe trauma, tracheal intubation and tracheostomy and urinary catheter had a significantly higher risk of developing HAI. We further investigated the effect of the length of catheter utilization time on the incidence of device-associated infections. Figure 1 shows that the incidence rate of device-associated infections increased with increasing catheter utilization time.

**Table 2:** Multivariate logistic regression analysis of potential risk factors for HAI in the ICU.

Comparison	*β*	*χ* ^2^	OR	95%CI	*P*
Age ≥ 60 years	0.014	0.014	1.014	0.804–1.279	0.907
Hospitalization over 2 weeks	1.663	212.304	5.276	4.192–6.641	<0.001
Antimicrobial prophylaxis	0.225	0.506	1.252	0.669–2.345	0.477
Chronic lung diseases	0.658	22.55	1.930	1.473–2.530	<0.001
Chronic heart diseases	0.354	4.102	1.424	1.015–2.000	0.043
Chronic renal diseases	0.944	16.547	2.569	1.644–4.016	<0.001
Current malignancy	0.653	6.497	1.921	1.177–3.137	0.011
Hypohepatia	0.542	6.168	1.719	1.128–2.619	0.013
Hypertension	0.265	3.697	1.303	0.995–1.708	0.055
Stroke	0.776	20.447	2.174	1.556–3.036	<0.001
Cerebral vascular accident	1.232	69.334	3.429	2.546–4.617	<0.001
Severe trauma	0.823	21.798	2.277	1.607–3.229	<0.001
Surgical intervention	0.478	2.062	1.613	0.853–3.049	0.151
Tracheal intubation and tracheostomy	0.897	33.366	2.453	1.790–3.360	<0.001
Urinary catheter	2.013	33.139	7.488	3.146–17.824	<0.001
CVC	0.146	0.857	1.157	0.849–1.575	0.356

HAI, hospital-acquired infection; ICU, intensive care unit; OR, odds ratio; CI, confidence interval; CVC, central venous catheter.

### 3.2 The effect of the adoption of an RT-NISS coupled with timely take corresponding comprehensive interventions on HAI prevention and control in the ICU

#### 3.2.1 Comparisons of the proportion of ICU patients receiving antibiotics and the proportion of HAI patients with pathogen detection in the ICU between 2021 and 2022

**Figure 2:**
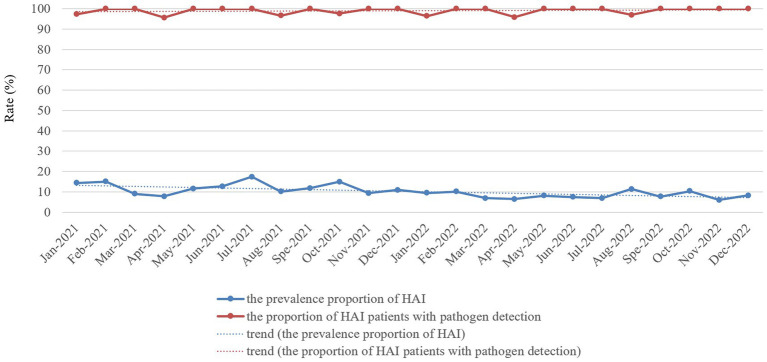
Monthly prevalence proportion of HAI cases and proportion of HAI patients with pathogen detection proportion in the ICU of HZFH from January 2021 to December 2022.

**Figure 3:**
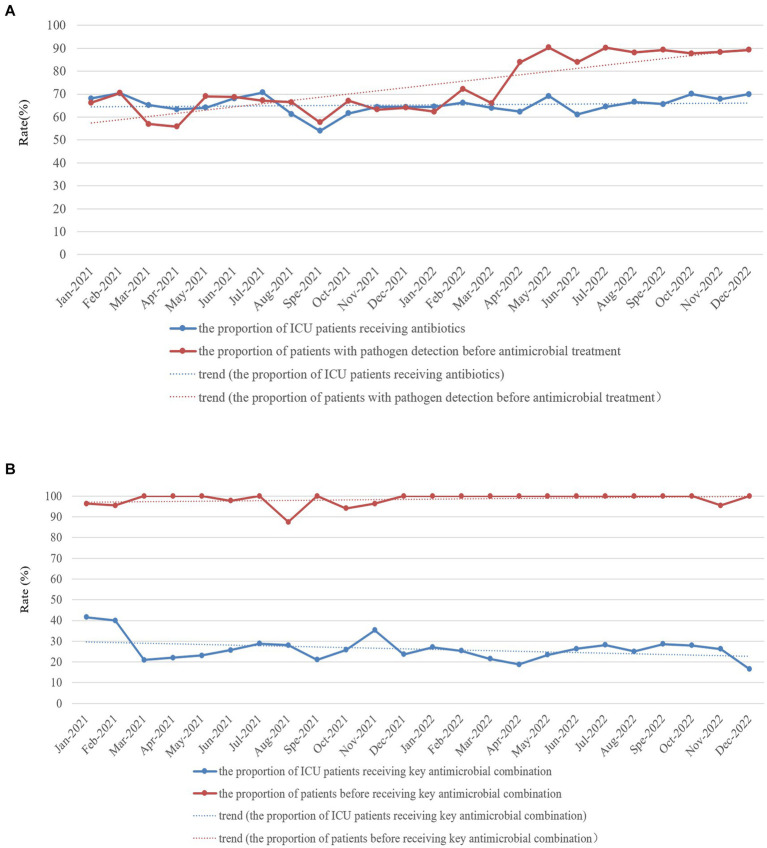
Monthly proportion of ICU patients receiving antibiotics **(A)** and key antimicrobial combinations **(B)** and proportion of patients with pathogen detection before antimicrobial **(A)** and key antimicrobial combination **(B)** treatment in the ICU of HZFH from January 2021 to December 2022.

The proportion of patients with pathogen detection was not significantly different between 2021 and 2022 (98.83% vs. 98.86%, χ^2^ = 0.000, *p* = 1.00), and the trend of the proportion of patients with pathogen detection from January 2021 to December 2022 remained stable (Figure 2). The proportion of ICU patients receiving antibiotics in 2021 was not significantly different from that in 2022 (63.32% vs. 65.44%, χ^2^ = 2.747, *p* = 0.097), whereas the proportion of patients with pathogen detection before antimicrobial treatment significantly increased in 2022 compared with the rate in 2021 (80.01% vs. 60.09%, χ^2^ = 172.701, *p* < 0.001). Furthermore, the trend of the proportion of ICU patients receiving antibiotics from January 2021 to December 2022 slightly increased (Figure 3A). In contrast, the trend of the proportion of patients with pathogen detection before antimicrobial treatment increased. There was no significant difference in the proportion of ICU patients receiving key antimicrobial combination between 2021 and 2022 (15.19% vs. 13.35%, χ^2^ = 3.368, *p* = 0.066); however, the proportion of patients with pathogen detection before receiving key antimicrobial combination significantly increased (92.54% vs. 97.80%, χ^2^ = 10.750, *p* = 0.001). The trend of key antimicrobial combinations from January 2021 to December 2022 slightly decreased, whereas the proportion of patients with pathogen detection before receiving key antimicrobial combinations in 2022 was stable at 100% (Figure 3B).

#### 3.2.2 Effect of an RT-NISS combined comprehensive interventions on hospital-acquired infection prevention and control in the ICU

We used an RT-NISS to identify 1706 patients >48 h after they were admitted to the ICU in 2021, including 342 (20.04%) HAI cases. This compares to 1769 patients identified >48 h after they were admitted to the ICU in 2022, including 265 (14.98%) HAI cases. The prevalence proportion of HAI in 2021 was significantly greater than that in 2022 (χ^2^ = 15.465, *p* < 0.001) and the monthly data in Figure 2 showed that the trend of the prevalence proportion of HAI from January 2021 to December 2022 was a slight decrease. For the specific HAI site data showed that 333 (19.52%) patients suffered respiratory infection, 97 (5.69%) UTI, 5 (0.29%) GTI, 8 (0.47%) SSI, 50 (2.93%) BSI, 9 (0.53%) abdominal infection, 3 (0.18%) encephalitis and 15 (0.88%) skin and soft tissue infection in 2021. And 202 (11.42%) patients suffered respiratory infection, 92 (5.20%) UTI, 0 (0%) GTI, 1 (0.06%) SSI, 29 (1.64%) BSI, 7 (0.40%) abdominal infection, 3 (0.17%) encephalitis, and 11 (0.62%) skin and soft tissue infection in 2022. With the exception of UTI, abdominal infection, encephalitis and skin and soft tissue infection, the prevalence proportions of different HAI sites (including respiratory infection, GTI, SSI, and BSI) in 2021 was significantly greater than those in 2022 (Table 3). Besides, the prevalence proportion of hospital-acquired MDROs was 9.14% (156 cases) in 2021 and 5.31% (94 cases) in 2022. The prevalence proportion of hospital-acquired MDROs in 2021 was significantly greater than that in 2022 (χ^2^ = 19.085, *p* < 0.001; Table 3).

**Table 3:** Comparative results of HAIs between 2021 and 2022 in the ICU.

Infection type	2021 (*n* = 1706)	2022 (*n* = 1769)	*χ* ^2^	*P*
HAIs cases (*n*/%)	342 (20.04)	265 (14.98)	15.465	<0.001
Respiratory infection	333 (19.52)	202 (11.42)	43.750	<0.001
UTI	97 (5.69)	92 (5.20)	0.397	0.528
GTI^*^	5 (0.29)	0 (0.00)		0.028
SSI^*^	8 (0.47)	1 (0.06)		0.019
BSI	50 (2.93)	29 (1.64)	6.520	0.011
Abdominal infection	9 (0.53)	7 (0.40)	0.329	0.566
Encephalitis^*^	3 (0.18)	3 (0.17)		1.000
Skin and soft tissue infection	15 (0.88)	11 (0.62)	0.775	0.379
Hospital-acquired MDROs (*n*/%)	156 (9.14)	94 (5.31)	19.085	<0.001

*Fisher’s exact test; HAI, hospital-acquired infection; ICU, intensive care unit; UTI, urinary tract infection; GTI, gastrointestinal tract infection; SSI, surgical site infection; BSI, bloodstream infection; MDRO, multidrug resistant organism.

**Figure 4:**
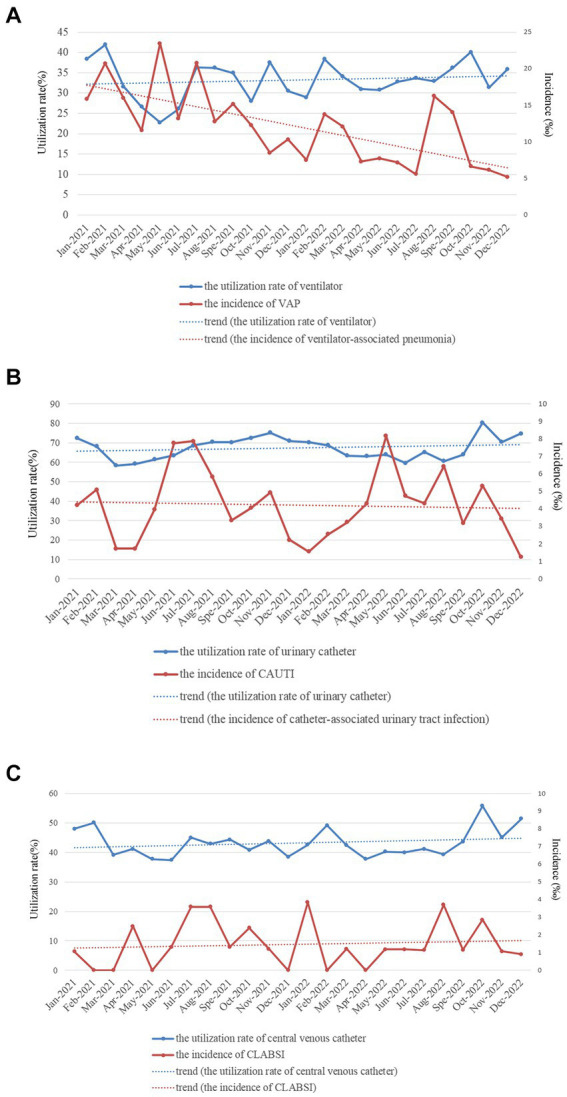
Monthly utilization rate of devices [**(A)** for ventilator, **(B)** for urinary catheter, and **(C)** for central venous catheter] and incidences of device-associated HAI infections **(A)** for ventilator-associated pneumonia, **(B)** for catheter-associated urinary tract infection, and **(C)** for central line-associated bloodstream infection in the ICU of HZFH from January 2021 to December 2022.

The utilization rate of ventilators in 2022 was significantly greater than that in 2021 (33.73% vs. 32.35%, χ^2^ = 10.226, *p* < 0.001), and the incidence of VAP in 2022 was lower than that in 2021 (15.02‰ vs. 9.19‰, χ^2^ = 17.627, *p* < 0.001). And the monthly data (Figure 4A) showed that the utilization rate of ventilators slightly increased, whereas the incidence rate of VAP significantly decreased from January 2021 to December 2022. The utilization rate of urinary catheters (67.45% vs. 66.85%, χ^2^ = 1.938, *p* = 0.164) and the incidence of CAUTI (4.44‰ vs. 4.02‰, χ^2^ = 1.380, *p* = 0.240) showed no significant difference between 2021 and 2022. And the monthly data (Figure 4B) showed that the utilization rate of urinary catheters and the incidence of CAUTI from January 2021 to December 2022 remained stable. The utilization rate of CVCs in 2022 was significantly greater than that in 2021 (43.89% vs. 42.28%, χ^2^ = 12.438, *p* < 0.001). In contrast, the incidence of central line-associated bloodstream infections was not significantly different between 2021 and 2022 (1.44‰ vs. 1.51‰, χ^2^ = 0.046, *p* = 0.831). And the monthly data (Figure 4C) showed that the utilization rate of central venous catheters from January 2021 to December 2022 slightly increased, whereas the incidence rate of central line-associated bloodstream infection remained stable.

All these findings demonstrated that the adoption of RT-NISS coupled with comprehensive interventions could prevent HAI events, thereby reducing its rate.

## 4 Discussion

HAIs are the leading cause of high mortality in the ICU (1, 2). In this study, we found that more ICU inpatients with HAI died than non-HAI inpatients (11.04% vs. 6.07%, χ^2^ = 19.180, *p* < 0.001). Furthermore, many studies have concluded that HAIs lead to prolonged hospital stays, long-term disability, and increased antimicrobial resistance, which will increase the financial burden and pain for inpatients (1–4). This is because many relative risk factors can lead to a significantly higher risk of developing HAI. Utilization of surveillance data in identifying risk is the primary purpose of HAI surveillance and the basis of corresponding good intervention-making for patients, which is an important strategy for nosocomial infection prevention and control (15). Studies on automatic electronic infection surveillance systems in ICU surveillance have been conducted in several countries, and those studies demonstrated that the sensitivity and specificity of HAI case ascertainment and time for HAI information and data collection can be improved by using automatic electronic infection surveillance systems (14, 15, 17–20). In the present study, we focused on determining the high risk of developing HAI in ICU patients using timely, adequate and accurate surveillance data from the RT-NISS. We used univariate and multivariate logistic regression analyses to analyze the surveillance data of the RT-NISS, and the results showed that hospitalization over 2 weeks, chronic lung diseases, chronic heart diseases, chronic renal diseases, current malignancy, hypohepatia, stroke, cerebral vascular accident, severe trauma, tracheal intubation and tracheostomy and utilization of urinary catheter were associated with a significantly higher risk of developing HAI in the ICU (Tables 2, 3). Furthermore, the study also showed that the incidence rates of device-associated infections increased with increasing catheter utilization time (Figure 1). These results are in line with Choudhuri’s study, which demonstrated that the length of catheter utility time and ICU stay were independent risk factors for HAIs in the ICU (21). All these demonstrated that daily assessment for possible transfer out of ICU or completion of catheter utilization according to the patient condition is necessary and essential for nosocomial infection prevention and control in the ICU. And for prevention and control of the device-associated HAIs in the ICU, real-time targeted surveillance of device-associated infections and timely feedback is essential. Furthermore, interventions include adequate hand hygiene, maximum sterile barrier and adequate training and examining healthcare workers are also indispensable.

Increasing the proportion of patients with pathogen detection of ICU patients was one of the critical infection prevention and control interventions implemented in 2022 in this study. Misuse, underuse, and prolonged antimicrobial treatment are the leading causes of MDROs (22, 23). Infection with MDROs affects treatment, prolongs the length of hospital stay, and even leads to death (24, 25). Therefore, clinicians should order timely pathogenic examinations for patients, select appropriate antibacterial treatment early and reduce or stop antibiotic therapy in a timely manner according to the patients’ infection condition and the results of pathogen detection and antimicrobial susceptibility tests. These measures will improve the quality of antimicrobial treatment of patients, avoid excessive exposure to antibacterial and reduce the risk of bacterial resistance so that patients with infections can receive appropriate treatment. Increasing pathogen detection can promote the rational use of antibiotics and detect potential infections in a timely manner. In recent years, the National Health Commission of the People’s Republic of China has paid particular attention to increase the rate of pathogenic examination of inpatients before antibiotic therapy and set it as the national medical quality and safety improvement goal. In this study, we set increasing the proportion of patients with pathogen detection of ICU inpatients as the central component of the infection prevention and control program. Furthermore, we implemented interventions such as monthly monitoring and reporting of proportion of ICU patients receiving antibiotics and proportion of patients with pathogen detection by the RT-NISS. Performance rewards or economic incentives were linked with the rational use of antibiotics and the execution of the proportion of patients with pathogen detection, as well as sufficient training in the rational use of antibiotics and pathogen detection knowledge for clinicians. The results of the present study showed that the proportion of patients with pathogen detection before antimicrobial treatment (80.01% vs. 60.09%, χ^2^ = 172.701, *p* < 0.001) and the proportion of patients with pathogen detection before receiving key antimicrobial combinations (97.80% vs. 92.54%, χ^2^ = 10.750, *p* = 0.001) significantly increased in 2022 compared with 2021. Consequently, the prevalence proportion of hospital-acquired MDROs significantly reduced in 2022 compared with 2021 (*χ*^2^ = 19.085, *p* < 0.001; Table 3).

Real-time automatic nosocomial infection surveillance alone will not result in successful surveillance, whereas using the data locally in a timely manner to improve the quality of care is the critical component of successful surveillance (20, 26). With the help of surveillance data captured in a timely, adequate and accurate manner by the RT-NISS, we could spend more time implementing infection prevention and control interventions rather than collecting surveillance data. Our analysis showed lower prevalence proportions of HAIs and specific infection sites, including respiratory infection, GTI, SSI, and BSI, in 2022 than in 2021. Target surveillance of the utilization rate of devices (ventilators, urinary catheters and CVCs) and the rate of device-associated HAIs (VAP, CAUTI, and CLABSI) was also a critical infection prevention and control intervention implemented in this study. Gozu’s study demonstrated that targeted surveillance of device-associated infections can reduce device-associated infections by 30% (27). Similarly, Gastmeier’s study presented a significant effect of surveillance in the reduction of CAUTI rates in the ICU (28). Here, we implemented some device-associated infection prevention and control interventions, including reducing unnecessary catheterization, daily assessment, removing devices as soon as possible according to the patient’s condition, training and examining healthcare workers (physicians and nurses) to ensure that they can master equipment instructions and operation, and specific prevention and control interventions (such as adequate hand hygiene, maximum sterile barrier, amongst others) were implemented before, during and after catheterization according to devices. The results of the present study showed that the incidence of VAP and CAUTI significantly decreased from 2021 to 2022 (15.02‰ vs. 9.19‰, χ^2^ = 17.627, *p* < 0.001) while the incidence of CAUTI (4.44‰ vs. 4.02‰, χ^2^ = 1.380, *p* = 0.240) and CLAB (1.44‰ vs. 1.51‰, χ^2^ = 0.046, *p* = 0.831) remained stable.

In conclusion, we analysed the ICU HAI case information collected adequately and accurately by an RT-NISS to identify several risk factors that were observed to be associated with a significantly high risk of developing HAI in ICU inpatients. These relative risk factors were hospitalization over 2 weeks, chronic lung diseases, chronic heart diseases, chronic renal diseases, current malignancy, hypohepatia, stroke, cerebral vascular accident, severe trauma, tracheal intubation and tracheostomy and urinary catheter. Furthermore, the adoption of the RT-NISS, together with comprehensive interventions, would reduce the risk and prevalence proportions of HAIs in the ICU.

## Data Availability

The raw data supporting the conclusions of this article will be made available by the authors, without undue reservation.

## References

[1] Chen YY Wang FD Liu CY Chou P. Incidence rate and variable cost of nosocomial infections in different types of intensive care units. Infect Control Hosp Epidemiol. (2009) 30:39–46. doi: 10.1086/592984, PMID: 19046058

[2] Osmon S Warren D Seiler SM Shannon W Fraser VJ Kollef MH. The influence of infection on hospital mortality for patients requiring > 48 h of intensive care. Chest. (2003) 124:1021–9. doi: 10.1378/chest.124.3.1021 12970033

[3] Rosenthal VD Jarvis WR Jamulitrat S Silva CP Ramachandran B Dueñas L. Socioeconomic impact on device-associated infections in pediatric intensive care units of 16 limited-resource countries: international nosocomial infection control consortium findings. Pediatr Crit Care Med. (2012) 13:399–406. doi: 10.1097/PCC.0b013e318238b260, PMID: 22596065

[4] Salgado E Bovera MM Rosenthal VD González H Pazmiño L Valencia F. Device-associated infection rates, mortality, length of stay and bacterial resistance in intensive care units in Ecuador: international nosocomial infection control Consortium's findings. World J Biol Chem. (2017) 8:95–101. doi: 10.4331/wjbc.v8.i1.95, PMID: 28289522 PMC5329718

[5] Zingg W Holmes A Dettenkofer M Goetting T Secci F Clack L. Hospital organization, management, and structure for prevention of health-care-associated infection: a systematic review and expert consensus. Lancet Infect Dis. (2015) 15:212–24. doi: 10.1016/S1473-3099(14)70854-0, PMID: 25467650

[6] Li Y Gong Z Lu Y Hu G Cai R Chen Z. Impact of nosocomial infections surveillance on nosocomial infection rates: a systematic review. Int J Surg. (2017) 42:164–9. doi: 10.1016/j.ijsu.2017.04.065, PMID: 28476543

[7] Gastmeier P. Nosocomial infection surveillance and control policies. Curr Opin Infect Dis. (2004) 17:295–301. doi: 10.1097/01.qco.0000136929.75543.8a 15241072

[8] Seifi A Dehghan-Nayeri N Rostamnia L Varaei S Akbari Sari A Haghani H. Health care-associated infection surveillance system in Iran: reporting and accuracy. Am J Infect Control. (2019) 47:951–5. doi: 10.1016/j.ajic.2018.12.028, PMID: 30738720

[9] Estan-Capell J Alarcon-Torres B Bermudez JD Martinez-Rodriguez L Martinez-Costa C. Effect of a surveillance system for decreasing neonatal nosocomial infections. Early Hum Dev. (2019) 131:36–40. doi: 10.1016/j.earlhumdev.2019.02.006 30825743

[10] Mingmei Du YX Suo J Liu B Jia N Huo R Chen C. Real-time automatic hospital-wide surveillance of nosocomial infections and outbreaks in a large Chinese tertiary hospital. BMC Med Inform Decis Mak. (2014) 14:8. doi: 10.1186/1472-6947-14-9 24475790 PMC3922693

[11] Sips ME Bonten MJM van Mourik MSM. Automated surveillance of healthcare-associated infections: state of the art. Curr Opin Infect Dis. (2017) 30:425–31. doi: 10.1097/QCO.0000000000000376 28505027

[12] Wen R Li X Liu T Lin G. Effect of a real-time automatic nosocomial infection surveillance system on hospital-acquired infection prevention and control. BMC Infect Dis. (2022) 22:857. doi: 10.1186/s12879-022-07873-7, PMID: 36384499 PMC9670380

[13] Villamarin-Bello B Uriel-Latorre B Fdez-Riverola F Sande-Meijide M Glez-Pena D. Gold standard evaluation of an automatic HAIs surveillance system. Biomed Res Int. (2019) 2019:1–10. doi: 10.1155/2019/1049575 PMC6778878 31662963

[14] Walter BC Koller AB Mandl H Adlassnig K-P. Electronic surveillance of healthcare-associated infections with MONI-ICU--a clinical breakthrough compared to conventional surveillance systems. Stud Health Technol Inform. (2010) 160:432–6. doi: 10.3233/978-1-60750-588-4-432 20841723

[15] Reilly JS McCoubrey J Cole S Khan A Cook B. Integrating intensive care unit (ICU) surveillance into an ICU clinical care electronic system. J Hosp Infect. (2015) 89:271–5. doi: 10.1016/j.jhin.2014.11.017 25601743

[16] TMoPH. The nosocomial infections diagnosis criterion. Natl Med J China. (2001).

[17] Freeman R Moore LSP García Álvarez L Charlett A Holmes A. Advances in electronic surveillance for healthcare-associated infections in the 21st century: a systematic review. J Hosp Infect. (2013) 84:106–19. doi: 10.1016/j.jhin.2012.11.031, PMID: 23648216

[18] de Bruin JS Adlassnig KP Blacky A Mandl H Fehre K Koller W. Effectiveness of an automated surveillance system for intensive care unit-acquired infections. J Am Med Inform Assoc. (2013) 20:369–72. doi: 10.1136/amiajnl-2012-000898, PMID: 22871398 PMC3638179

[19] De Bus L Diet G Gadeyne B Leroux-Roels I Claeys G Steurbaut K. Validity analysis of a unique infection surveillance system in the intensive care unit by analysis of a data warehouse built through a workflow-integrated software application. J Hosp Infect. (2014) 87:159–64. doi: 10.1016/j.jhin.2014.03.010, PMID: 24856115

[20] Schaumburg T Kohler N Breitenstein Y Kolbe-Busch S Hasenclever D Chaberny IF. ICU infection surveillance can be based on electronic routine data: results of a case study. BMC Infect Dis. (2023) 23:126. doi: 10.1186/s12879-023-08082-6, PMID: 36859254 PMC9979400

[21] Choudhuri A Chakravarty M Uppal R. Epidemiology and characteristics of nosocomial infections in critically ill patients in a tertiary care intensive care unit of northern India. Saudi J Anaesth. (2017) 11:402–7. doi: 10.4103/sja.SJA_230_17, PMID: 29033719 PMC5637415

[22] Arnoldo L Smaniotto C Celotto D Brunelli L Cocconi R Tignonsini D. Monitoring healthcare-associated infections and antimicrobial use at regional level through repeated point prevalence surveys: what can be learnt? J Hosp Infect. (2019) 101:447–54. doi: 10.1016/j.jhin.2018.12.016, PMID: 30597175

[23] Ramachandran P Rachuri NK Martha S Shakthivel R Gundala A Battu TS. Implications of over prescription of antibiotics: a cross-sectional study. J Pharm Bioallied Sci. (2019) 11:S434–7. doi: 10.4103/JPBS.JPBS_62_19, PMID: 31198382 PMC6555336

[24] Lin J Gao X Cui Y Sun W Shen Y Shi Q. Increased multidrug resistant isolates: new clinical burdens for 66 hospitals in Shanghai, 2015 to 2017. Ann Transl Med. (2020) 8:112. doi: 10.21037/atm.2019.12.91, PMID: 32175405 PMC7049002

[25] Qureshi S Maria N Zeeshan M Irfan S Qamar FN. Prevalence and risk factors associated with multi-drug resistant organisms (MDRO) carriage among pediatric patients at the time of admission in a tertiary care hospital of a developing country. Cross Sect Study BMC Infect Dis. (2021) 21:547. doi: 10.1186/s12879-021-06275-5, PMID: 34107903 PMC8191205

[26] Woeltje KF Lin MY Klompas M Wright MO Zuccotti G Trick WE. Data requirements for electronic surveillance of healthcare-associated infections. Infect Control Hosp Epidemiol. (2014) 35:1083–91. doi: 10.1086/677623 25111915

[27] Gozu A Clay C Younus F. Hospital-wide reduction in central line-associated bloodstream infections_ a tale of two small community hospitals. Infect Control Hosp Epidemiol. (2011) 32:619–22. doi: 10.1086/660098, PMID: 21558777

[28] Gastmeier P Behnke M Schwab F Geffers C. Benchmarking of urinary tract infection rates: experiences from the intensive care unit component of the German national nosocomial infections surveillance system. J Hosp Infect. (2011) 78:41–4. doi: 10.1016/j.jhin.2011.01.021, PMID: 21481490

